# Recommendations for Methodology of Virtual Reality Clinical Trials in Health Care by an International Working Group: Iterative Study

**DOI:** 10.2196/11973

**Published:** 2019-01-31

**Authors:** Brandon Birckhead, Carine Khalil, Xiaoyu Liu, Samuel Conovitz, Albert Rizzo, Itai Danovitch, Kim Bullock, Brennan Spiegel

**Affiliations:** 1 Division of Health Services Research Department of Medicine Cedars-Sinai Health System Los Angeles, CA United States; 2 Laboratoire Interdisciplinaire de Recherche Appliquée en Économie de la Santé Paris Descartes University Paris France; 3 Department of Health Policy and Management Fielding School of Public Health University of California, Los Angeles Los Angeles, CA United States; 4 Institute for Creative Technologies University of Southern California Los Angeles, CA United States; 5 Department of Psychiatry Cedars-Sinai Health System Los Angeles, CA United States; 6 Department of Psychiatry Stanford University School of Medicine Palo Alto, CA United States

**Keywords:** clinical trials, consensus, virtual reality

## Abstract

**Background:**

Therapeutic virtual reality (VR) has emerged as an efficacious treatment modality for a wide range of health conditions. However, despite encouraging outcomes from early stage research, a consensus for the best way to develop and evaluate VR treatments within a scientific framework is needed.

**Objective:**

We aimed to develop a methodological framework with input from an international working group in order to guide the design, implementation, analysis, interpretation, and communication of trials that develop and test VR treatments.

**Methods:**

A group of 21 international experts was recruited based on their contributions to the VR literature. The resulting Virtual Reality Clinical Outcomes Research Experts held iterative meetings to seek consensus on best practices for the development and testing of VR treatments.

**Results:**

The interactions were transcribed, and key themes were identified to develop a scientific framework in order to support best practices in methodology of clinical VR trials. Using the Food and Drug Administration Phase I-III pharmacotherapy model as guidance, a framework emerged to support three phases of VR clinical study designs—VR1, VR2, and VR3. VR1 studies focus on content development by working with patients and providers through the principles of human-centered design. VR2 trials conduct early testing with a focus on feasibility, acceptability, tolerability, and initial clinical efficacy. VR3 trials are randomized, controlled studies that evaluate efficacy against a control condition. Best practice recommendations for each trial were provided.

**Conclusions:**

Patients, providers, payers, and regulators should consider this best practice framework when assessing the validity of VR treatments.

## Introduction

Therapeutic virtual reality (VR) is an innovative treatment modality to manage a broad range of health conditions and is gaining considerable attention [1-19]. Users of VR wear a head-mounted display (HMD) with a close-proximity screen that creates a sense of being transported into life-like, three-dimensional worlds. VR has been used to assess and treat a wide variety of medical, surgical, psychiatric, and neurocognitive conditions including pain [1,2,4,9,13,18], addiction [20-25], anxiety disorders [3,6,7,14-15,26-34], schizophrenia [10,11,19,35-38], eating disorders [1,8,39-45], stroke rehabilitation [5,12,16-17,45-47], vestibular disorders [48], and movement disorders [49]. One of the first published uses of HMD-based therapy was the treatment of acrophobia in 1995 [50]. There have also been functional magnetic resonance imaging studies demonstrating the effect of VR on the brain during receipt of a painful stimuli [51,52]. VR is thought to work through a combination of distraction, extinction learning, cognitive-behavioral principles, mindful meditation, stress reduction, gate-control theory, and the spotlight theory of attention [53,54]. Importantly, VR has become increasingly portable, immersive, and vivid, which has enabled the technology to be used in a broad range of inpatient and outpatient applications.

As the use of therapeutic VR expands, it is essential that guidelines are established to ensure scientific rigor in the development and evaluation of VR applications, similar to established standards for pharmacotherapies [30,55]. VR developers would benefit from systematic guidance on best practices for designing and conducting VR clinical trials. To fulfil this unmet need, we garnered input from an international working group, called the Virtual Reality Clinical Outcomes Research Experts (VR-CORE) committee. This paper presents the resulting best practice framework informed by expert input, along with specific recommendations on ways to conduct high-quality VR treatment trials. Although the focus of this paper is VR, the framework also applies to other emerging “XR” technologies, including augmented reality and mixed reality, as the methodologic considerations for clinical trials are largely similar across XR platforms.

## Methods

### Identifying Virtual Reality Clinical Outcomes Research Experts

We performed a systematic review of randomized controlled trials (RCTs) using therapeutic VR to help identify eligible VR-CORE committee members through review of author lists. To cover the largest breadth of studies, the literature search focused on existing meta-analyses of therapeutic VR RCTs identified through search of PubMed, Google Scholar, and the Cochrane Database of Systematic Reviews using a combination of keywords: (“virtual reality” OR “VR”) AND (“review [pt]” OR “systematic review [pt]” OR “meta-anal*” OR “metaanaly*”). Based on our literature search, and supplemented by recommendations from established experts, we developed a multidisciplinary group for the VR-CORE, including experts in fields relevant to developing and testing VR treatments such as user-centered design principles, software design, epidemiology, statistics, and clinical trial methodology. The committee was formulated to balance expertise across clinical disciplines (medicine, pediatrics, surgery, psychology, psychiatry, neuroscience, anesthesia, nursing, and rehabilitation) and reflect multinational perspectives.

### Collecting Input From the Virtual Reality Clinical Outcomes Research Experts

To obtain systematic feedback from the committee, a series of electronic meetings were held to collect and synthesize structured input. An iterative approach was modeled after similar processes were employed by our previous working groups in other fields of health care [56,57]. Using an online meeting platform that allows users to view and react to each other’s comments [58], committee members initially responded to open-ended “think aloud” prompts [59] (eg, “When you think about the current state of the clinical VR research, what comes to your mind?”), followed by increasingly specific probes prepared by the moderators (eg, “What should be the role of human centered design principles in developing VR treatments?”). The full set of questions and responses is listed in Multimedia Appendix 1. The active members of the VR-CORE at the time of this discussion are listed in the Acknowledgments section. Emergent themes and proposed methodologic best practices were culled from the online dialogue, and the resulting recommendations were distributed to the members for synthesis and iterative rephrasing.

## Results

### Emergent Themes from Virtual Reality Clinical Outcomes Research Experts Meetings

Multimedia Appendix 1 provides excerpted transcripts of the VR-CORE responses to discussion topics. Key themes drawn from the online dialogue are summarized in the following sections.

#### Perceptions Regarding the Current State of Clinical Virtual Reality Research

Committee members described the current state of clinical VR research as the “Wild West” with a “lack of clear guidelines and standards.” The state of current VR research was described as “heterogeneous,” often focused “more on the tech rather than the theories behind it.” Committee members expressed concern that much of the current research is “merely descriptive” in nature, often insufficiently powered, focused on small case reports and retrospective analyses, and often does not employ experimental designs.

#### Perceptions About Ways to Improve Virtual Reality Literature

The committee believed it is vital to “include the patients’ voice early and often in the development of VR treatments” and that developers must “carefully, systematically, and meticulously seek the patients’ feedback” through participatory research and design thinking that involves multidisciplinary collaboration. The committee acknowledged the importance of including the voice of providers as well. The committee also called for better definitions and standardization of therapeutic VR study designs.

#### Most Important Considerations for Designing and Standardizing Clinical Virtual Reality Trials

The committee described various stages for developing and validating VR treatments, beginning with content development in partnership with end-users, progressing through initial clinical testing and safety evaluation, and ending with properly powered RCTs. The committee outlined a wide range of considerations for each stage (Multimedia Appendix 1), including the importance of standardizing control groups, selecting clinically relevant outcome measures, reporting which equipment was used in the trial, accounting for dropouts and disqualified participants, and allowing for pragmatic features of each study design.

### Clinical Trial Framework of the Virtual Reality Clinical Outcomes Research Experts

#### The Framework

Although there are fundamental best practices in study design that apply to all biomedical intervention trials, the committee identified VR-specific attributes that are unique considerations for VR trials. Using the Food and Drug Administration Phase I-III pharmacotherapy model as guidance [55] and combining the results of literature synthesis with VR-CORE input, a framework emerged to support three phases of VR clinical study designs, named VR1, VR2, and VR3.

**Figure 1:**
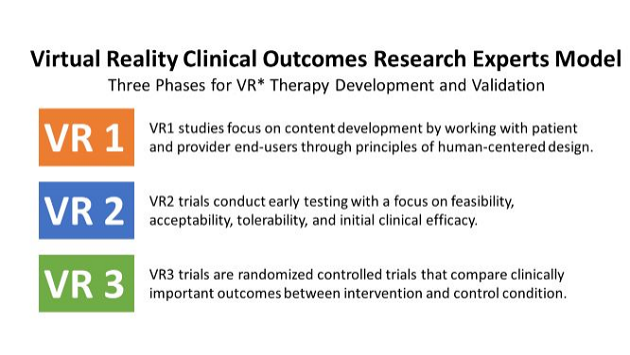
Summary of VR1, VR2, VR3. VR: virtual reality.

VR1 studies focus on content development by working with patient and provider end-users through principles of human-centered design. VR2 trials conduct early testing with a focus on feasibility, acceptability, tolerability, and initial clinical efficacy. VR3 trials are RCTs that compare clinically important outcomes between intervention groups and a control condition. Each study should undergo ethical review before initiation. Figure 1 summarizes each phase of the VR-CORE model. Best practice recommendations for each trial design are described below.

#### VR1 Studies

The committee strongly believes that therapeutic VR applications should be designed with direct input from patient and provider end-users. Lack of patient involvement, poor requirement definitions, and nonadaptation to user feedback are some of the common factors that explain failures of digital interventions [60]. Incorporating patients into the design process enables developers to increase the relevance and effectiveness of VR treatments. The committee stresses that VR treatments should be created with acknowledgment of patients’ knowledge, attitudes, beliefs, preferences, and expectations of therapeutic VR. VR-CORE refers to a VR1 study as one that results in the development of VR treatment in partnership with patient and provider end-users and follows best practices for patient-centered design.

After their review of the literature on human-centered design both generally [61,62] and in relation to digital [60] and VR interventions [63], the committee identified three key principles that are fundamental for developing “desirable, feasible and viable” VR treatments [61]. These principles promote empathy, team collaboration, and continuous user feedback (Table 1). The committee believes that the use of these principles allows development teams to better identify users’ needs, incorporate user feedback, and institute rapid cycle improvements that generate more relevant products at lower cost [64]. The key principles for VR1 studies are outlined in Table 1.

**Table 1:** Summary of design principles, strategies, and recommended best practices for VR1 studies.

Design principles and strategies		Best practices
**Inspiration through empathizing**		
	Recruitment	Determine the population of interest (who do we need to hear from?). Think about a variety of factors (age, gender, ethnicity, health conditions, and social position).
	Observation	Learn about patients and their behavior by observing them in a clinically relevant context. Observe what patients do in a specific context and what they see and say.
	Patient interviews	Perform individual cognitive interviews and focus groups with patients to learn about their relevant needs, struggles, experiences, fears, aspirations, and expectations. Document a diverse set of opinions from a variety of patient profiles across ages (eg, above vs below “digital divide”), comorbidities, and experience and comfort with technology (eg, technophiles vs technophobes).
	Expert interviews	Perform cognitive interviews and focus groups with relevant experts representing different points of view such as treating providers and other staff members.
	Journey mapping and personas	Define the patient user and describe the sequence of events in which the patient will experience the virtual reality treatment within the context of their illness experience.
**Ideation through team collaboration**		
	Sharing stories and notes	Collect stories, pictures, impressions, and notes about patients’ experiences and behavior. Share information among team members to generate many ideas through techniques such as storyboarding, storytelling, and mind mapping.
	Generating ideas	Encourage team members to generate ambitious ideas without being judged. The committee believes that idea generation should be distinguished from idea evaluation. After generating ideas, the team evaluates each idea and culls out the most feasible and appropriate idea for prototyping within technical and budgetary constraints.
**Prototyping through continuous user feedback**		
	Building prototype	Convert ideas into tangible figures through drawings or mock-ups and obtain initial user feedback prior to advanced prototyping. Iteratively improve designs with user feedback.
	Continuously testing prototype	Test quickly and iterate on the design of the prototype by collecting both positive and negative user feedback. Document all stages of user feedback in the resulting VR1 study paper.

##### The Design Process of Virtual Reality Treatments Should Promote Empathy

The committee believes that the more attuned a development team is to the specific perspective and needs of patients, the more likely they are to design meaningful VR treatments. Promoting empathy toward the design process involves carefully listening to and elucidating patients’ social environment, needs, fears, desires, habits, hopes, aspirations, and expectations. The committee recommends initiating the design process with an *inspiration step, or exercise focused on culling* patients’ voice and understanding their needs, struggles, and experiences. Table 1 describes best practices for sparking inspiration within the framework of empathy. Different patient profiles and scenarios should be included in this first step. Many techniques can be used to develop empathy and inspiration of the design team. These include qualitative assessments, observations, spending time with users, and conducting interviews and user experiments. In addition, a patient journey map can be used to illustrate the interpretation of a story from a patient’s perspective. The working group also recommends seeking input from relevant nonpatient end-users, including health care providers who may prescribe the VR treatment or interact with patient users.

##### The Design Process of Virtual Reality Treatments Should Promote Team Collaboration

The committee believes that team collaboration is fundamental for collectively designing a VR treatment and synthesizing data collected during the inspiration step. *Brainstorming* helps generate ideas from the initial corpus of data and findings. Table 1 describes best practices for ideation within the framework of team collaboration. The process of ideation allows team members to think expansively and divergently. As a range of ideas is generated, some ideas will be extreme or ambitious, whereas others will be achievable. Depending on the time and the available budget, the team decides what ideas should be prototyped further.

#### The Design Process of Virtual Reality Treatments Should Promote Continuous User Feedback

An effective VR treatment should be developed through continuous user feedback and *iterative prototyping*, thereby enabling the team to rapidly test their ideas during real-time assessment from end-users. Table 1 describes best practices for VR treatment prototyping within the framework of user feedback. Prototypes should be refined with continuous testing by patient end-users, and failures are viewed as a way to learn and improve the prototype to better meet users’ needs. Hence, the number of defects will tend to be lower and less costly in the future. To help facilitate the learning process for patients, it is recommended, when feasible, that the research team use a “mirroring” program [65] to allow the research staff to see what the patient is viewing through the VR headset and help them learn the user interface.

Briefly, the committee believes that the VR1 treatment design process should start with end-users. VR-CORE recommends specifying who the real users are and what they say, see, feel, and do. Hence, implementation of a patient-design approach is an important way to place users at the center of the VR design process. For researchers who are developing an open-source VR intervention that they would like to share with the academic community for collaborative V1 development process, the use of a software-development platform such as GitHub.com [66] and citation of the latest version of the program within the methods section of VR1 research papers are recommended. The committee also recommends use of the Integrate, Design, Assess, and Share checklist developed by Mummah and colleagues [60] as a supplemental, structured guide for conducting a VR1 study.

#### VR2 Trials

Once the research team has developed a VR treatment in partnership with end-users, the resulting product should undergo initial assessment in the target patient population within a representative clinical setting, herein termed a VR2 trial. Modeled after the work of Mosadeghi and colleagues [67], the purpose of VR2 trials is to conduct early testing with a focus on *acceptability*, *feasibility*, *tolerability*, and *initial clinical efficacy* prior to initiating a more definitive VR3 clinical trial. Although developers may opt to bypass a VR2 trial in lieu of a VR3 trial, there is a risk of subjecting an incompletely tested intervention to a larger and costlier RCT, and best practices in digital intervention development suggest an intermediary stage between initial VR design and definitive testing [60]. The following sections describe the features of a VR2 trial.

##### Clinical Setting

In contrast to a VR1 study, which is focused on collaborative content development in a design environment, the VR2 trial evaluates what happens when the VR treatment is placed in the hands of target patients within the intended clinical setting. For example, a VR treatment focused on management of inpatient pain should be tested in an inpatient environment. A VR treatment targeting outpatient stroke rehabilitation should be evaluated in locations where patients receive rehabilitation, such as in a physical therapy center or, if intended, at home. In short, a comprehensive VR2 trial evaluates the VR treatment in the natural setting(s) where the product is intended to be used. Table 2 summarizes the best practices for VR2.

##### Acceptability

In the context of a VR2 trial, *acceptability* refers to a patient’s willingness to use the VR treatment. Previous research on therapeutic VR reveals a drop off in the relation between patient eligibility to receive VR and patient willingness to try VR [67]. The disconnect emphasizes that many patients are uninterested in using novel health technologies such as VR, particularly when hospitalized or under duress. Among those who are eligible for a VR trial, some choose not to participate for a wide variety of reasons. Patients may express varying degrees of skepticism, fear, vulnerability, and concern regarding psychological consequences or simply not want to be bothered by the equipment [67]. In a VR2 trial, investigators collect data regarding patient willingness to try the VR treatment, including reasons why they did or did not find the intervention to be acceptable for use. Researchers should collect and report acceptability data using techniques such as focus groups, cognitive interviews, or structured questionnaires.

##### Feasibility

In the context of a VR2 trial, *feasibility* is the degree to which the VR treatment can be successfully integrated within the flow of usual care. The committee noted that even the best designed VR treatments can face implementation challenges when applied on the front lines of health care delivery [67]. It is wise for developers to understand potential barriers early and often, identify workarounds and solutions to these barriers, and only then consider testing their interventions in VR3 RCT trials. For example, patients and providers often seek information regarding the frequency and “dosing” of a VR treatment; these details could be manually collected in the context of a VR2 trial. Similarly, treatments deployed in a clinical environment may be unfamiliar to doctors, nurses, and other health care providers, giving researchers an opportunity to study the interaction among staff and proactively identify areas of confusion or misuse. The committee recommends including a table that enumerates patient, provider, technical, and operational barriers to use; identifies root causes; and offers solutions to enhance effectiveness in future clinical applications.

##### Tolerability

The VR2 trial offers an early opportunity to evaluate patient *tolerability* of the VR treatment, including both hardware and software components. Researchers should measure and report the prevalence of patient-reported physical (eg, vertigo, nausea, and “cybersickness”) and emotional (eg, fear and anxiety) adverse effects of the VR treatment, along with any discomfort or inconvenience related to the VR equipment (eg, ill-fitting headset, facial or nasal pain, inability to explore the three-dimensional environment fully due to limited mobility).

Cybersickness (or VR sickness) is a unique side effect of VR. There a several different terms used interchangeably within the literature, such as simulator sickness or “sim sickness,” although some believe they are different types of motion sickness [68]. When the vestibular system and oculomotor system notice a discrepancy between reality and the virtual environment, one or more of following symptoms ensue: eyestrain, nausea, fatigue, headache, blurred vision, and postural instability [69]. The specific mechanism of cybersickness is still unknown. 

**Table 2:** Summary of best practice recommendations for VR2 trials.

Trial attribute	Best practice
Patient population	Study a representative population for whom the VR^a^ treatment is intended. Recruit a large enough sample to represent the breadth and depth of target patients and provide statistically stable estimates in descriptive analytics.
Clinical setting	Select a clinical setting that represents the intended environment for the VR treatment to be used (eg, inpatient vs outpatient, clinic vs home based)
Assessment of acceptability	Collect data regarding patient willingness to try the VR treatment, including reasons why they did, or did not, find the intervention to be acceptable for use. Researchers should collect and report acceptability data using techniques such as focus groups, cognitive interviews, or structured questionnaires.
Assessment of feasibility	Conduct patient and provider interviews to identify potential barriers and facilitators to using the VR treatment in the intended clinical environment. Collect information regarding the optimal frequency and “dosing” of a VR treatment; consider manualizing these details, where possible. Study interactions among staff and proactively identify areas of confusion or misuse. Consider including a table that enumerates patient, provider, technical, and operational barriers to use; identifies root causes; and offers solutions to enhance effectiveness in future clinical applications.
Assessment of tolerability	Measure and report the prevalence of patient-reported physical and emotional adverse effects of the VR treatment, along with any discomfort or inconvenience related to the VR equipment.
Assessment of initial clinical efficacy	Identify and justify selection of a clinically relevant and validated PRO^b^ to evaluate the evidence of efficacy. Measure the PRO before and after receipt of the VR treatment; consider comparing results against nonrandomized concurrent or retrospective control groups, where available.

^a^VR: virtual reality.

^b^PRO: patient-reported outcome.

Recommendations for developers already exist [70,71]: appropriately accelerate within the program [71,72], anticipate changes in direction [73], affect changes in the field of view [73], establish realistic virtual avatar movements, reduce drops in the frame rate below 60 fps [71], blur the display with movement [74], and provide other solutions at the level of program design. 

There are also several strategies for medical staff and researchers including habituation [75], assessment of the risk of side effects before the intervention [76], use of oculomotor exercises before the intervention [77], and diaphragmatic breathing during the intervention [78]. One of the most useful strategies is to limit the total duration of each treatment session, particularly early in the process [70]. 

The VR-CORE recommends assessing for side effects at every phase (VR1, VR2, and VR3). Regarding assessment scales, the Simulator Sickness Questionnaire is the most commonly used scale in the literature [70,72,75,76].

##### Initial Clinical Efficacy

Although the VR2 trial is not designed to definitively test whether a VR treatment is efficacious or effective, it offers an early opportunity to measure efficacy within the context of a small clinical trial. There is no requirement in a VR2 trial to include a control group, although uncontrolled case series carry a higher risk of bias than controlled studies; even studies with nonrandomized concurrent controls, “wait list” controls, or retrospective controls may reduce the risk of bias as compared to an uncontrolled series.

Regardless of the inclusion of a control group, investigators should identify a clinically relevant and validated patient- reported outcome (PRO) to evaluate the evidence of efficacy. For example, a study evaluating pain might include a standard 11-point numeric rating scale [79] before and after exposure to the VR treatment. A study evaluating stroke rehabilitation might measure physical function with the National Institutes of Health Patient Reported Outcomes Measurement Information System [80]. Selection of the most appropriate PRO is at the discretion of the research team, but should be carefully justified and capture the most salient features of patient-reported health that might improve with the VR treatment.

#### VR3 Trials

The most definitive clinical validation of a VR treatment is the VR3 trial, which is a prospective, adequately powered, methodologically rigorous RCT evaluating clinical outcomes and safety in target patients receiving the VR treatment as compared to a control condition. Although the therapeutic mechanism of action may be studied as a secondary goal in a VR3 trial (eg, through neuroimaging, blood biomarkers, and physiologic testing), the principal goal is to evaluate the treatment’s impact on a clinically meaningful patient outcome rather than surrogate markers.

Although the committee acknowledged understandable costs and resource barriers involved in conducting VR3 trials, there was broad agreement that RCTs are of equal scientific importance in therapeutic VR as any other form of treatment and should be prioritized whenever possible. Multicenter collaborations may facilitate VR3 trials by combining patients and resources through shared protocols. The features of a VR3 trials are described below and summarized in Table 3.

##### Standardization of Intervention and Patient Population

Having been developed in a VR1 study and initially tested in a VR2 trial, the study intervention should be clearly described in preparation for a VR3 trial. Researchers should provide details regarding the equipment used; visualizations employed (with representative screenshots or videos); and frequency, duration, and timing of use. Optimally, the intervention should be manualized, and at the very least, enough details should be provided to allow other investigators to repeat the trial, if desired. The Template for Intervention Description and Replication checklist provides a useful framework for describing study interventions [81] and should be applied to VR treatments. The target patient population should be clearly described, including explicit inclusion and exclusion criteria employed. Certain exclusion criteria may be standardized among VR trials, such as a history of significant motion sickness, active nausea, and vomiting or epilepsy.

##### Selection of Control Condition

The committee acknowledged that there is no perfect or standardized control condition for all VR treatment trials; the optimal control depends on the patient population, proposed mechanism of action of the intervention, and clinical setting, among other considerations. Selection of the control is at the discretion of the research team but should be justified and explained. The committee described a hierarchy of control conditions, ranging from “usual care” without any active intervention to passive visualizations on a two-dimensional screen and nonimmersive visualizations within a headset, immersive but passive experiences within a headset, and immersive and active experiences within a headset. Selection of the optimal control may be guided by considering the hypothesized target of engagement and the proposed mechanism of action.

##### Randomization

Randomization should be described and ideally achieved using an appropriate computer program (eg, MS Excel Random Number Generator) [81] or random number tables without involvement of the investigators who enrolled the patients.

##### Blinding and Concealment of Allocation

The committee acknowledged that blinding and concealment can be challenging, but they identified techniques to incorporate these RCT principles within the constraints of VR research. For example, Spiegel and colleagues (2017) achieved concealment of allocation in an RCT comparing a library of VR content to a “health and wellness” television channel in hospitalized patients experiencing pain [83]. At the time of consent, the researchers explained to patients that the study compared “two different audiovisual experiences designed to reduce pain,” but did not describe the details of the competing interventions. Patients randomized to the television intervention did not know that VR was the other condition and vice versa. This approach may reduce the “novelty effect” of receiving VR rather than a familiar experience like television. Equipoise may also be achieved by exposing patients in both arms to headsets, but varying the content viewed within the headset (eg, immersive vs nonimmersive, active vs passive). At a minimum, study analysts should be blinded to patient group allocation, allowing for unbiased evaluation of the data without the knowledge of the study group. Patients should be asked not to reveal details of the program they experienced to decrease the chance of unblinding the study analysts. The measurement of perceived group assignment at the end of the study can help assess the success of blinding within the study. This should be done at the discretion of the research team.

##### Endpoints

Like the VR2 trial, VR3 trials must prespecify a clinically relevant and validated PRO as the primary endpoint. The study must be appropriately powered to demonstrate a minimally clinically important difference (MCID) [84] in that endpoint between the VR treatment and control arms. The psychometrics of PRO measurement are beyond the scope of this document, but existing references may assist investigators in protocol development [84,85]. Secondary endpoints may include a variety of clinical, imaging, biometric, and physiologic surrogate markers, as deemed appropriate by the study team. Like VR2 trials, potential adverse events must be prospectively measured and reported.

##### Study Duration

VR3 studies should monitor patients for a sufficient period to determine whether the VR treatment meaningfully impacts clinically important outcomes. One-time, short-term evaluations may be insufficient to evaluate the true clinical value of an intervention. Follow-up over several days may be appropriate if the study only focuses on hospital stay, but measurement over weeks, or even months, may be necessary to assess the impact on long-term clinical benefits.

##### Presentation and Analysis of Results

VR-CORE recommends that the primary outcome be reported as the before and after *difference in difference* between study arms, with accompanying 95% CIs. For example, the change in the mean PRO score before and after the VR intervention should be compared against the change in the mean PRO score before and after the control intervention. In addition, the panel recommends predefining a binary response criterion, guided by the MCID of the primary endpoint. The proportion achieving the MCID should be reported and compared between groups, and the resulting number needed to treat should be calculated.

**Table 3:** Summary of best practice recommendations for VR3 Trials.

Trial attribute	Best practices
Patient population	Study a representative population for whom the VR^a^ treatment is intended. The target patient population should be clearly described, including explicit inclusion and exclusion criteria employed.
Clinical setting	Select a clinical setting that represents the intended environment for the VR treatment to be used (eg, inpatient vs outpatient, clinic vs home based).
Standardizing intervention	Provide details regarding the equipment used; visualizations employed; and frequency, duration, and timing of use for VR treatment. Consider following the TIDIER^b^ checklist [81] as a useful framework for describing features of the VR treatment.
Selecting control condition	Select and justify the control condition(s) by considering the hypothesized target of engagement and the proposed mechanism of action.
Randomization	Randomization should be achieved using an appropriate computer program (eg, MS Excel Random Number Generator) [82] or random number tables without involvement of the investigators who enrolled the patients.
Blinding and concealment of allocation	Describe efforts to conceal allocation of the study intervention to the participants. Describe efforts to blind patient, providers, and analysts, wherever possible. Measure perceived group assignment to assess success of blinding.
Endpoints	Prespecify a clinically relevant and validated PRO^c^ as the primary endpoint. The psychometric properties of available PRO measures may need to be modified in the context of immersive therapy and then revalidated as needed. Trials must be appropriately powered to demonstrate an MCID^d^ [83] in the primary endpoint between the VR treatment and control arms. Secondary endpoints may include a variety of clinical, imaging, biometric, and physiologic surrogate markers, as deemed appropriate by the study team. Potential adverse events must be prospectively measured and reported.
Study duration	Select and justify the follow-up period that is sufficient to determine whether the VR treatment meaningfully impacts clinically important outcomes.
Presentation and analysis of results	Report the before and after *difference in difference* in the primary outcome measure between study arms, with accompanying 95% CIs. Predefine a binary response criterion, guided by the MCID of the primary endpoint. The proportion achieving the MCID should be reported and compared between groups, and the resulting number needed to treat should be calculated. Use intention-to-treat analysis for primary outcome assessment. Per-protocol analysis may be reported if prespecified, as relevant. To perform a multivariable analysis, it is optimal to have at least 10 (preferably, 20) observations for each independent variable included in the multivariable model.
Reporting the trial	Trial must be registered on a publicly accessible registry (eg, clinicaltrials.gov). All completed trials should be published, whether positive or negative. The CONSORT^e^ guidelines provide the framework for reporting RCTs [86] and should be followed in VR3 trials. Include a CONSORT diagram demonstrating the flow of patients through each stage of the trial, including the number screened to the number randomized into each study group and the number analyzed.

^a^VR: virtual reality.

^b^TIDIER: Template for Intervention Description and Replication.

^c^PRO: patient-reported outcome.

^d^MCID: minimally clinically important difference.

^e^CONSORT: Consolidated Standards for Reporting Trials

The primary analyses should use the intention-to-treat population, including all patients randomized regardless of follow-up or receipt of study interventions. However, per-protocol analysis may be appropriate in certain situation, such as if patients refuse the VR treatment after randomization; in this instance, reporting the rate of refusal would be important, but investigators might also seek to compare therapeutic responses only among those receiving the intervention.

Multivariable analysis may be useful in adjusting for prespecified confounding factors (especially if not equally distributed in the study groups) and exploring independent predictors of outcomes. To perform a multivariable analysis, it is optimal to have at least 10 (preferably, 20) observations for each independent variable included in the multivariable model.

##### Trial Reporting

VR3 trials must be registered in a publicly accessible registry (eg, such as ClinicalTrials.gov). All completed trials should be published, regardless of whether they are positive or negative. The Consolidated Standards for Reporting Trials (CONSORT) guidelines provide the framework for reporting RCTs [86] and should be followed in VR3 trials. VR3 trials must include a CONSORT diagram to demonstrate the flow of patients through each stage of the trial, including the number screened to the number randomized into each study group and the number analyzed.

#### Conclusions

To improve methodological quality in the therapeutic VR literature, the VR-CORE international working group presents a three-part framework for best practices in developing and testing VR treatments. This framework may be used to facilitate development of high-quality, effective, and safe VR treatments that meaningfully improve patient outcomes. Patients, providers, payers, and regulators should consider this framework when assessing the validity of VR treatments.

## Appendix

Multimedia Appendix 1 Excerpted transcripts of Virtual Reality Committee of Outcomes Research Experts responses to selected discussion topics. Key themes and phraseology included in the manuscript are highlighted. Note that not all committee members responded to all questions.

## Abbreviations

CONSORT: Consolidated Standards for Reporting Trials
HMD: head-mounted display
MCID: minimally clinically important difference
PRO: patient-reported outcome
RCT: randomized controlled trial
TIDIER: Template for Intervention Description and Replication
VR: virtual reality
VR-CORE: Virtual Reality Clinical Outcomes Research Experts

## References

[1] Dascal J, Reid M, IsHak WW, Spiegel B, Recacho J, Rosen B, Danovitch I (2017). Virtual Reality and Medical Inpatients: A Systematic Review of Randomized, Controlled Trials. Innov Clin Neurosci.

[2] Mahrer NE, Gold JI (2009). The use of virtual reality for pain control: a review. Curr Pain Headache Rep.

[3] Valmaggia LR, Latif L, Kempton MJ, Rus-Calafell M (2016). Virtual reality in the psychological treatment for mental health problems: An systematic review of recent evidence. Psychiatry Res.

[4] Wismeijer AAJ, Vingerhoets AJJM (2005). The use of virtual reality and audiovisual eyeglass systems as adjunct analgesic techniques: a review of the literature. Ann Behav Med.

[5] Lohse KR, Hilderman CGE, Cheung KL, Tatla S, Van der Loos HFM (2014). Virtual reality therapy for adults post-stroke: a systematic review and meta-analysis exploring virtual environments and commercial games in therapy. PLoS One.

[6] Botella C, Serrano B, Baños RM, Garcia-Palacios A (2015). Virtual reality exposure-based therapy for the treatment of post-traumatic stress disorder: a review of its efficacy, the adequacy of the treatment protocol, and its acceptability. Neuropsychiatr Dis Treat.

[7] Gonçalves R, Pedrozo AL, Coutinho ESF, Figueira I, Ventura P (2012). Efficacy of virtual reality exposure therapy in the treatment of PTSD: a systematic review. PLoS One.

[8] de Carvalho MR, Dias TRDS, Duchesne M, Nardi AE, Appolinario JC (2017). Virtual Reality as a Promising Strategy in the Assessment and Treatment of Bulimia Nervosa and Binge Eating Disorder: A Systematic Review. Behav Sci (Basel).

[9] Chirico A, Lucidi F, De Laurentiis M, Milanese C, Napoli A, Giordano A (2016). Virtual Reality in Health System: Beyond Entertainment. A Mini-Review on the Efficacy of VR During Cancer Treatment. J Cell Physiol.

[10] da Costa RMEM, de Carvalho LAV (2004). The acceptance of virtual reality devices for cognitive rehabilitation: a report of positive results with schizophrenia. Comput Methods Programs Biomed.

[11] Macedo M, Marques A, Queirós C (2015). Virtual reality in assessment and treatment of schizophrenia: a systematic review. J Bras Psiquiatr.

[12] Luque-Moreno C, Ferragut-Garcías A, Rodríguez-Blanco C, Heredia-Rizo AM, Oliva-Pascual-Vaca J, Kiper P, Oliva-Pascual-Vaca A (2015). A Decade of Progress Using Virtual Reality for Poststroke Lower Extremity Rehabilitation: Systematic Review of the Intervention Methods. Biomed Res Int.

[13] Malloy KM, Milling LS (2010). The effectiveness of virtual reality distraction for pain reduction: a systematic review. Clin Psychol Rev.

[14] Parsons TD, Rizzo AA (2008). Affective outcomes of virtual reality exposure therapy for anxiety and specific phobias: a meta-analysis. J Behav Ther Exp Psychiatry.

[15] Powers MB, Emmelkamp PMG (2008). Virtual reality exposure therapy for anxiety disorders: A meta-analysis. J Anxiety Disord.

[16] Rodrigues-Baroni JM, Nascimento LR, Ada L, Teixeira-Salmela LF (2014). Walking training associated with virtual reality-based training increases walking speed of individuals with chronic stroke: systematic review with meta-analysis. Braz J Phys Ther.

[17] de Rooij IJM, van de Port IGL, Meijer JG (2016). Effect of Virtual Reality Training on Balance and Gait Ability in Patients With Stroke: Systematic Review and Meta-Analysis. Phys Ther.

[18] Triberti S, Repetto C, Riva G (2014). Psychological factors influencing the effectiveness of virtual reality-based analgesia: a systematic review. Cyberpsychol Behav Soc Netw.

[19] Rus-Calafell M, Garety P, Sason E, Craig TJK, Valmaggia LR (2018). Virtual reality in the assessment and treatment of psychosis: a systematic review of its utility, acceptability and effectiveness. Psychol Med.

[20] Girard B, Turcotte V, Bouchard S, Girard B (2009). Crushing virtual cigarettes reduces tobacco addiction and treatment discontinuation. Cyberpsychol Behav.

[21] Kuntze MF, Stoermer R, Mager R, Roessler A, Mueller-Spahn F, Bullinger AH (2001). Immersive virtual environments in cue exposure. Cyberpsychol Behav.

[22] Bordnick PS, Traylor AC, Carter BL, Graap KM (2012). A Feasibility Study of Virtual Reality-Based Coping Skills Training for Nicotine Dependence. Res Soc Work Pract.

[23] Carter BL, Bordnick P, Traylor A, Day SX, Paris M (2008). Location and longing: the nicotine craving experience in virtual reality. Drug Alcohol Depend.

[24] Thompson-Lake DGY, Cooper KN, Mahoney JJ, Bordnick PS, Salas R, Kosten TR, Dani JA, De La Garza R (2015). Withdrawal Symptoms and Nicotine Dependence Severity Predict Virtual Reality Craving in Cigarette-Deprived Smokers. Nicotine Tob Res.

[25] Bordnick PS, Traylor A, Copp HL, Graap KM, Carter B, Ferrer M, Walton AP (2008). Assessing reactivity to virtual reality alcohol based cues. Addict Behav.

[26] Maples-Keller JL, Price M, Rauch S, Gerardi M, Rothbaum BO (2017). Investigating Relationships Between PTSD Symptom Clusters Within Virtual Reality Exposure Therapy for OEF/OIF Veterans. Behav Ther.

[27] Maples-Keller JL, Bunnell BE, Kim S, Rothbaum BO (2017). The Use of Virtual Reality Technology in the Treatment of Anxiety and Other Psychiatric Disorders. Harv Rev Psychiatry.

[28] Meyerbröker K, Emmelkamp PMG (2010). Virtual reality exposure therapy in anxiety disorders: a systematic review of process-and-outcome studies. Depress Anxiety.

[29] Emmelkamp PMG, Krijn M, Hulsbosch AM, de Vries S, Schuemie MJ, van der Mast CA (2002). Virtual reality treatment versus exposure in vivo: a comparative evaluation in acrophobia. Behav Res Ther.

[30] Bouchard S, Dumoulin S, Robillard G, Guitard T, Klinger E, Forget H, Loranger C, Roucaut FX (2017). Virtual reality compared with exposure in the treatment of social anxiety disorder: a three-arm randomised controlled trial. Br J Psychiatry.

[31] Morina N, Ijntema H, Meyerbröker K, Emmelkamp PM (2015). Can virtual reality exposure therapy gains be generalized to real-life? A meta-analysis of studies applying behavioral assessments. Behav Res Ther.

[32] Rizzo AA, Difede J, Rothbaum BO, Johnston S, McLay RN, Reger G, Gahm G, Parsons T, Graap K, Pair J (2009). VR PTSD exposure therapy results with active duty OIF/OEF combatants. Stud Health Technol Inform.

[33] Maples-Keller JL, Yasinski C, Manjin N, Rothbaum BO (2017). Virtual Reality-Enhanced Extinction of Phobias and Post-Traumatic Stress. Neurotherapeutics.

[34] Difede J, Cukor J, Jayasinghe N, Patt I, Jedel S, Spielman L, Giosan C, Hoffman HG (2007). Virtual reality exposure therapy for the treatment of posttraumatic stress disorder following September 11, 2001. J Clin Psychiatry.

[35] Freeman D (2008). Studying and treating schizophrenia using virtual reality: a new paradigm. Schizophr Bull.

[36] Kim K, Kim J, Kim J, Park D, Jang HJ, Ku J, Kim C, Kim IY, Kim SI (2007). Characteristics of social perception assessed in schizophrenia using virtual reality. Cyberpsychol Behav.

[37] Sorkin A, Weinshall D, Modai I, Peled A (2006). Improving the accuracy of the diagnosis of schizophrenia by means of virtual reality. Am J Psychiatry.

[38] Veling W, Pot-Kolder R, Counotte J, van Os J, van der Gaag M (2016). Environmental Social Stress, Paranoia and Psychosis Liability: A Virtual Reality Study. Schizophr Bull.

[39] Ferrer-Garcia M, Gutiérrez-Maldonado J, Riva G (2013). Virtual Reality Based Treatments in Eating Disorders and Obesity: A Review. J Contemp Psychother.

[40] Cesa GL, Manzoni GM, Bacchetta M, Castelnuovo G, Conti S, Gaggioli A, Mantovani F, Molinari E, Cárdenas-López G, Riva G (2013). Virtual reality for enhancing the cognitive behavioral treatment of obesity with binge eating disorder: randomized controlled study with one-year follow-up. J Med Internet Res.

[41] Riva G (2011). The key to unlocking the virtual body: virtual reality in the treatment of obesity and eating disorders. J Diabetes Sci Technol.

[42] Gutiérrez-Maldonado J, Ferrer-García M, Caqueo-Urízar A, Moreno E (2010). Body image in eating disorders: the influence of exposure to virtual-reality environments. Cyberpsychol Behav Soc Netw.

[43] Ferrer-García M, Gutiérrez-Maldonado J (2012). The use of virtual reality in the study, assessment, and treatment of body image in eating disorders and nonclinical samples: a review of the literature. Body Image.

[44] Marco JH, Perpiñá C, Botella C (2013). Effectiveness of cognitive behavioral therapy supported by virtual reality in the treatment of body image in eating disorders: one year follow-up. Psychiatry Res.

[45] Gutiérrez-Maldonado J, Pla-Sanjuanelo J, Ferrer-García M (2016). Cue-exposure software for the treatment of bulimia nervosa and binge eating disorder. Psicothema.

[46] Subramanian SK, Lourenço CB, Chilingaryan G, Sveistrup H, Levin MF (2013). Arm motor recovery using a virtual reality intervention in chronic stroke: randomized control trial. Neurorehabil Neural Repair.

[47] Crosbie JH, Lennon S, McGoldrick MC, McNeill MDJ, McDonough SM (2012). Virtual reality in the rehabilitation of the arm after hemiplegic stroke: a randomized controlled pilot study. Clin Rehabil.

[48] Bergeron M, Lortie CL, Guitton MJ (2015). Use of Virtual Reality Tools for Vestibular Disorders Rehabilitation: A Comprehensive Analysis. Adv Med.

[49] You SH, Jang SH, Kim Y, Kwon Y, Barrow I, Hallett M (2005). Cortical reorganization induced by virtual reality therapy in a child with hemiparetic cerebral palsy. Dev Med Child Neurol.

[50] Rothbaum BO, Hodges LF, Kooper R, Opdyke D, Williford JS, North M (1995). Effectiveness of computer-generated (virtual reality) graded exposure in the treatment of acrophobia. Am J Psychiatry.

[51] Hoffman HG, Richards TL, Bills AR, Van Oostrom T, Magula J, Seibel EJ, Sharar SR (2006). Using FMRI to study the neural correlates of virtual reality analgesia. CNS Spectr.

[52] Hoffman HG, Richards TL, Coda B, Bills AR, Blough D, Richards AL, Sharar SR (2004). Modulation of thermal pain-related brain activity with virtual reality: evidence from fMRI. Neuroreport.

[53] Li A, Montaño Z, Chen VJ, Gold JI (2011). Virtual reality and pain management: current trends and future directions. Pain Manag.

[54] McCaul KD, Malott JM (1984). Distraction and coping with pain. Psychol Bull.

[55] U.S Food & Drug Administration.

[56] Laine L, Spiegel B, Rostom A, Moayyedi P, Kuipers EJ, Bardou M, Sung J, Barkun AN (2010). Methodology for randomized trials of patients with nonvariceal upper gastrointestinal bleeding: recommendations from an international consensus conference. Am J Gastroenterol.

[57] Grade Working Group.

[58] Slack.

[59] Ericsson AKS, Simon H (1993). Protocol Analysis: Verbal Reports as Data.

[60] Mummah SA, Robinson TN, King AC, Gardner CD, Sutton S (2016). IDEAS (Integrate, Design, Assess, and Share): A Framework and Toolkit of Strategies for the Development of More Effective Digital Interventions to Change Health Behavior. J Med Internet Res.

[61] (2015). Field Guide to Human-Centered Design.

[62] Brown T (2008). Design thinking. Harv Bus Rev.

[63] Jerald J (2015). The VR Book: Human-centered Design For Virtual Reality.

[64] Carr S, Halliday A, King A, Liedtka J, Lockwood T (2010). The Influence of Design Thinking in Businessome Preliminary Observations. Design Management Review.

[65] (2017). Oculus.

[66] Github.

[67] Mosadeghi S, Reid MW, Martinez B, Rosen BT, Spiegel BMR (2016). Feasibility of an Immersive Virtual Reality Intervention for Hospitalized Patients: An Observational Cohort Study. JMIR Ment Health.

[68] Stanney KM, Kennedy RS, Drexler JM (1997). Cybersickness is not simulator sickness.

[69] Bruck S, Watters PA (2009). Estimating cybersickness of simulated motion using the simulator sickness questionnaire (SSQ): A controlled study.

[70] Porcino TM, Clua E, Trevisan D, Vasconcelos CN, Valente L (2017). Minimizing cyber sickness in head mounted display systems: design guidelines and applications.

[71] Yao R, Heath T, Davies A, Forsyth T, Mitchell N, Hoberman P (2014). Oculus.

[72] Mazloumi Gavgani A, Hodgson DM, Nalivaiko E (2017). Effects of visual flow direction on signs and symptoms of cybersickness. PLoS One.

[73] Lin J, Abi-Rached H, Lahav M (2004). Virtual guiding avatar: An effective procedure to reduce simulator sickness in virtual environments. Proceedings of the SIGCHI conference on Human factors in computing systems.

[74] Budhiraja P, Miller MR, Modi AK, Forsyth D (2017). ARXIV.

[75] Gavgani AM, Nesbitt KV, Blackmore KL, Nalivaiko E (2017). Profiling subjective symptoms and autonomic changes associated with cybersickness. Auton Neurosci.

[76] Tyrrell R, Sarig-Bahat H, Williams K, Williams G, Treleaven J (2017). Simulator sickness in patients with neck pain and vestibular pathology during virtual reality tasks. Virtual Reality.

[77] Park WD, Kim YS, Jang SW, Kim GA, Kim YH, Son W (2017). A study on cyber sickness reduction by oculo-motor exercise performed immediately prior to viewing virtual reality (VR) content on head mounted display (HMD). JVE Journals.

[78] Russell MEB, Hoffman B, Stromberg S, Carlson CR (2014). Use of controlled diaphragmatic breathing for the management of motion sickness in a virtual reality environment. Appl Psychophysiol Biofeedback.

[79] Dworkin RH, Turk DC, Farrar JT, Haythornthwaite JA, Jensen MP, Katz NP, Kerns RD, Stucki G, Allen RR, Bellamy N, Carr DB, Chandler J, Cowan P, Dionne R, Galer BS, Hertz S, Jadad AR, Kramer LD, Manning DC, Martin S, McCormick CG, McDermott MP, McGrath P, Quessy S, Rappaport BA, Robbins W, Robinson JP, Rothman M, Royal MA, Simon L, Stauffer JW, Stein W, Tollett J, Wernicke J, Witter J, IMMPACT (2005). Core outcome measures for chronic pain clinical trials: IMMPACT recommendations. Pain.

[80] Cella D, Yount S, Rothrock N, Gershon R, Cook K, Reeve B, Ader D, Fries JF, Bruce B, Rose M, PROMIS Cooperative Group (2007). The Patient-Reported Outcomes Measurement Information System (PROMIS): progress of an NIH Roadmap cooperative group during its first two years. Med Care.

[81] Hoffmann TC, Glasziou PP, Boutron I, Milne R, Perera R, Moher D, Altman DG, Barbour V, Macdonald H, Johnston M, Lamb SE, Dixon-Woods M, McCulloch P, Wyatt JC, Chan A, Michie S (2014). Better reporting of interventions: template for intervention description and replication (TIDieR) checklist and guide. BMJ.

[82] Saghaei Mahmoud (2011). An overview of randomization and minimization programs for randomized clinical trials. J Med Signals Sens.

[83] Tashjian VC, Mosadeghi S, Howard AR, Lopez M, Dupuy T, Reid M, Martinez B, Ahmed S, Dailey F, Robbins K, Rosen B, Fuller G, Danovitch I, IsHak W, Spiegel B (2017). Virtual Reality for Management of Pain in Hospitalized Patients: Results of a Controlled Trial. JMIR Ment Health.

[84] Jaeschke R, Singer J, Guyatt GH (1989). Measurement of health status. Ascertaining the minimal clinically important difference. Control Clin Trials.

[85] Norman GR, Sloan JA, Wyrwich KW (2003). Interpretation of changes in health-related quality of life: the remarkable universality of half a standard deviation. Med Care.

[86] Curran PJ, Bauer DJ (2011). The disaggregation of within-person and between-person effects in longitudinal models of change. Annu Rev Psychol.

[87] Begg C, Cho M, Eastwood S, Horton R, Moher D, Olkin I, Pitkin R, Rennie D, Schulz KF, Simel D, Stroup DF (1996). Improving the quality of reporting of randomized controlled trials. The CONSORT statement. JAMA.

