# Supplementary material for: Recommendations for Methodology of Virtual Reality Clinical Trials in Health Care by an International Working Group: Iterative Study
Source: JMIR Ment Health. 2019 Jan 31;6(1):e11973. doi: 10.2196/11973 (PMC6374734; doi:10.2196/11973)
Supplement: Multimedia Appendix 1 [file mental_v6i1e11973_app1.pdf]

Supplemental information:

Appendix A. Excerpted transcripts of VR-CORE responses to selected discussion topics. Key themes and phraseology included in manuscript are highlighted. Note that not all committee members responded to all questions.

When you think about the current state of clinical VR research what comes to mind?

- Wild west
- So much opportunity and potential with little guidance.
- A lot of opportunities, a lot of interest from patients, very little knowledge of what is required technically or in terms of content.
- Sometimes there is a rush to demonstrate clinical efficacy without taking the time to develop the intervention from the ground up.
- It's a bit of the wild west. Software is evolving, hardware is evolving, new indications and possible applications are emerging. These new applications are not limited to therapeutics--they include education, training, and theory. Investigations are proliferating rapidly, with new investigative groups having varied research experience becoming involved. Studies are highly heterogeneous. Altogether, when I think of "the current state of academic VR" I think of a nascent field with imminent growth potential. Very exciting.
- The very first is the lack of clear guidelines and standards. This can be felt even more when you look into certain topics such as age limits, psychological considerations, and ethics. I would say that… we focus more on the tech rather than the applications and the theories behind it.
- First, I would say that there are not enough studies using both experimental and longitudinal designs. Many of the studies that I have seen are merely descriptive. We need more longitudinal studies in healthcare that show that this technology is making a long-term impact.
- The majority of research that I have read fall into 3 buckets: the very small, sort of case report, description style; the retrospective review, and a few number of prospective RCTs. Given the young field, I believe the technology is moving faster than the research, and subsequently it is being deployed in novel ways that may not be actually beneficial to patients since it has not been verified in that setting to be beneficial
- I think the field is maturing but struggling to make real headway due to rapid changes in technology. There is lack of consensus regarding definition and such diverse programs and applications. There are so many different programs it is hard to come to consensus about the efficacy of the intervention.
- I agree that a VR-only arm is generally necessary

What areas of the literature need improvement? What improvements are necessary?

- All areas need improvement… especially randomized controlled trials needed especially in behavioral health outside of anxiety.
- Would like to see more inclusion of consumer voice in this field as well. Is this something that consumers actively want (I think so but we don't have that data) although we are forging ahead regardless
- In some subfields of computer science, including patients and consumers (HCI: human-computer interaction) has become standard practice, and can extend from the very early stages of development (participatory design) to so-called user testing & clinical studies.
- It is vital to include the patients' voice early and often in the development of VR treatments. It makes no sense to develop a patient-facing intervention without carefully, systematically, and meticulously seeking the patients' feedback. Also, if a doctor will be involved in administering a VR treatment, then the doctors (or other clinicians) need to be involved too when designing the intervention.
- Basic definitions are needed. There is a need to define the features of VR hardware and software. There is a need to determine best experimental designs. Methods to appropriately control for confounding. For many subjects the novelty of VR may generate a placebo response, which must be further explored and understood. Interventions need to be standardized and manualized. The role of clinicians needs to be explored--can they serve as guides, therapists, coaches that help patients integrate and learn from the VR experience?
- As I always tell others, we are forming the foundations of future educational and clinical practices of immersive technologies. We have to do it right.
- We need clarity on the steps required to develop a valid VR treatment. Should outline those steps and provide a sort of checklist for developers to consider when constructing and testing their intervention.
- Agreed that the novelty/prestige aspect of VR producing placebo effects is very important. An earlier thread mentioned the dearth of longitudinal studies which also applies here.
- VR experiences are inherently different depending on the users and environment. Most VR studies lack standardization of the study population and the controls. For example, we have studied VR with extremely tight inclusion/exclusion criteria based on some retrospective data that has proven that those who are most apt to benefit from VR are most likely to utilize it.
- I agree basic definitions, especially on VR hardware and software are needed. It is important to categorize the type of studies. Although design thinking is not common practice in our healthcare system, I think this is key to develop new VR solutions for patients. It will be great if we can come up with specific guidelines/suggestions in this area, and can agree on publication of such patient centered design studies.

- Agree regarding need for better definitions. I also think that there needs to be better cooperation in order to get big projects happening. There are many small studies - it also seems that many studies are conducted as student projects so limited size, time, budget.
- Need more feasibility studies.
- I think we need to develop some clear ethics guidelines that can be universally accepted. Secondly, I am mostly asked about the cost of technology in the study.
- Collaboration between different disciplines. Need to consider translation - what is the key message for clinicians. Small, underpowered evaluations of custom VR programs which are not accessible to clinicians have limited utility.

What do you think are the most important issues to consider when designing a clinical VR study?

- How to design a control? How to fund this with least amount of conflicts of interests? How to design an intervention that won't be obsolete at the end of the study due to the rapid technological advances?
- How to interface with software designers to ensure a quality product that is appropriate for clinical use. It has been like learning a new language and I still don't feel fluent in this. I almost need a glossary of VR terms to support clinical study design and communication with software developers. As mentioned before - user experience is important not just in the tech sense but also in the sense of incorporating consumers as valued partners in this process
- Finding technical collaborators who are willing -- and able -- to invest in learning about what's required in the health domain, and to take the time to communicate on both sides (technical & medical). It requires trust & time. However, with the risk of sounding too Polyanna about it, there *are* a handful of technical experts who are pretty devoted to contributing in the health domain, and have been doing so for quite some time. If we could find a way to get them *and* their long-time health experts/collaborators to develop a way to share what they they've learned, in a way that can be disseminated (maybe on levels of intro onwards), we could make much more significant progress. We have interest. We have willingness. The industry is doing what they think they can do to help developers grow a consumer base, but they're often scared away from the health domain because of legal fears and fear of FDA requirements.
- Because VR appears to be safe, low cost, relatively easy to implement intervention, it is important to be flexible and open new applications. At the same time, for any particular indication or application, it is important to being to strengthen the quality of studies (see my comment on specific design considerations above) in order to minimize bias and maximize generalizability of findings.

- Our biggest concerns in the pediatric population is continuing to allow and about assent from children, ensuring children will not have any adverse reactions such as a seizure or nausea, and perhaps the most interesting ethical dilemma - the formation of false memories. Children may not be able to distinguish between fact and fiction and we must ensure children do not have an unpleasant experience that they recall as reality. This requires a lot of vetting of the library of experiences.
- Things I consider: intended purpose of the VR program, patient population, work flow of the clinical setting for intervention, assessments tools that will be used in the study. How can the study design be improved from prior studies? How will data be collected?
- Who are the participants. What defines them (a diagnosis, a symptom, a setting, a limitation, a simulation or learning need)? What is the control? How will blinding and randomization be achieved? How will treatment fidelity be established? What will be the measure of change? How many participants are needed? What type of analysis will be conducted (intent to treat)?
- How to account for drop-outs/disqualified participants
- How to control for the VR experience itself without taking all control away from the user. For example, it is not uncommon for providers to work with multiple tools when providing care for a patient and allow for the patient to pick which of the agreed upon medicine, exercises, etc works best for them. Within VR, we believe that a certain amount of flexibility will allow maximum return. Thus, we have bucketed VR experiences in our last study into 'active' vs 'passive' experiences and are letting our study participants choose what content they would like to experience within the active library or passive library…Active meaning actually participating in a game (like shooting something) vs passive meaning just looking around (like sitting on a beach)

What areas of clinical VR research do you think need to be standardized across fields, if any?

- Standardized Controls for different aspects and mechanisms of VR
- Efficacious treatments need to be standardized in order to disseminate effectively
- What I would insist on, however, is definition what is meant by VR (some PubMed papers, for example, that are purportedly about VR don't involve VR at all (no stereoscopic displays, no HMDs, no spatialized sound, and so on). I'd also insist on the authors of PubMed papers to actually list the specific equipment they use because it makes a difference.
- Standards of research quality: ie: what are the features of a low, medium, or high quality VR study. How do research standards that have been applied in other fields best apply to VR? Also there is a pressing opportunity to validate rating scales and measures in VR.
- Also at least a portion of the exclusion criteria could be standardized for almost any trial using a head mounted display (Hx of motion sickness, seizures, etc).

- One thing I like to see is having a psychologists on the research team, at least at the time of study design, to make sure we are not hurting participants in any possible way. A set of standard criteria for choosing participants seems useful to me
- We have been aggressively trying to determine which previously validated scales - usually in the realm of procedural compliance, anxiety, or pain - are most applicable to VR research. Either these previously validated scales shall be used consistently across all VR studies, or, probably better would be for modifications of these scales to be validated in VR patients and the modified scales be used. Once we have validated instruments, the research can focus on different populations and different VR experiences.
- Reporting. Use of the TIDIER checklist for intervention reporting. Use of the CONSORT for RCTs.

When testing a new clinical VR intervention, what considerations do you have about selecting a proper control intervention?

- How do you control for the hardware? How do you control for the software? How do you control for the novelty of the experience? How do you blind the subject and the investigator to the subjects allocation?
- There are many considerations for selecting a control, but it is hard to standardize an optimal control. I don't think we can be too proscriptive in our recommendations here, because the optimal control varies considerably depending on the proposed mechanism of action of the VR treatment, the study population, and the clinical environment. For some studies, "usual care" might be the control. For others, it might be an active control. The active control could be all sorts of things, ranging from a 2D experience mimicking the 3D VR experience, another 2D experience altogether thought to have a similar mechanism of action as the VR experience, or whatever. The point is, it's hard to standardize this other than to provide general guidance and suggestions to consider.
- It seems one could either control for hardware or software, but challenging to do both. To control software one could put the same program on a tablet PC. If one wanted to control for hardware they could make a recorded version of the intervention.
- In some cases I would work on different levels of immersion. As an example, I know that some hardware companies have two versions of goggles: VR and HM video players. So if your usual intervention is a video or images (e.g. distraction) then you have to similar goggles one with video/image (control) and the other with VR.
- The novelty of the experience is not necessarily something that needs to be controlled against. We know that novelty plays into therapies all the time. For example, if we use a tablet to show a video or project the same video on the wall, the novelty of the wall projected video wins every time. Our trials have included passive VR experience vs no goggles, active vs passive, and active vs no goggles. We did consider, and perhaps should have included a blindfold vs passive as a control group. Some believe that by just not

watching, this is part of the effect, so by doing a blindfolded vs passive experience, you can get more to the point of whether it was the experience itself or just not watching. We consider a passive experience to be the least level of distraction by just allowing patient to look around - they are not moving in the experience and not actively doing anything except looking. The next level would be an experience where you are not moving by perhaps shooting at something. The next level is you are moving (like sledding down a hill) plus you are actively doing something (like collecting coins). By creating a hierarchy of immersivity, you can start to bucket experiences into these categories and then start to set population studies against different categories.

- Depends on research questions. Dose is important.
- What is the hypothesized target of engagement.
- Tricky. I'm not sure anyone has thought that through to the degree we'd like. It seems to range from VR-NoVR (nothing, such as sitting quietly in a room) to various media forms (from non-interactive, like watching a movie or listening to audio to interactive forms, like a screen-based video game) to watching an interaction in a VR simulation that someone else did (replaying that "fly thru"). Where does one draw this line? I think taking "the hypothesized target of engagement" into account is crucial to answer this question because differing targets will result in differing VR "content" -- and its control.

What thoughts do you have about optimally standardizing a VR intervention during a clinical trial?

- This should only be done on RCT… early trials should be flexible so we can explore and keep up with changing technology.
- I think it's important to think about the mechanism(s) of action of the intervention. Is it the whole experience, the specific content, a specific aspect of the experience, the dose or duration? In other words, what are the active ingredients of the VR experience? The goal of standardization shouldn't be to constrain or limit VR, but rather to improve the ability of investigators to evaluate specific components in a consistent manner.
- The intervention should be clearly described. Authors should list the equipment used and provide screenshots or even videos of the visualizations, themselves. The frequency, duration, and timing of exposure should be described in the methods section of the manuscript.
- The intervention should be "manualized" to the degree that is possible. I realize that is sometimes hard to achieve at the testing phase, and sometimes manualization cannot occur until more is known about the timing and frequency of use. But in order for the study to be reproducible, which is a defining characteristic of an RCT, the VR3 trial, in particular, should endeavor to present a "manualized" intervention, or at the very least, provide enough details so someone else can try to reproduce the study.

- I believe this is a question of practicality vs rigid research design. I think that categorizing the experiences based on degree of user participation is one way to bridge the gap between practicality and rigid research design. Obviously if everyone used the same experience with the same instrument to measure effect, it would be the most rigid, but this lacks practicality and transferrability across institutions in some incidences. By creating a set of guidelines that can categorize the experiences, this may lead to easier way for researchers to publish results that translated by other users. Plus, it may allow some flexibility for users to choose their experience within a set of parameters. The comparison group may just be standard of care (ie, no VR) or head to head against other categories of experiences.
- Again depends if the research is examining tailored interventions versus standardised interventions. Researchers should make available their protocols/manuals and discuss how fidelity was assessed.

What should clinical VR trialists consider when selecting endpoints for their studies?

- Outcome measures from more traditional interventions to compare.
- Essential that we ground this in traditional research in order to truly compare between different approaches.
- Important to use a validated, clinically relevant, patient-reported outcome (PRO) as the primary endpoint of a VR treatment trial. Surrogate endpoints like physiologic outcomes, imaging outcomes, etc, may be very important to understand mechanism, but to understand if patients actually get better, we need to ask them. That requires using an appropriate PRO that has been "validated" in various ways (face validity, content validity, construct validity, criterion validity – basic psychometric requirements of any PRO).
- Does it work better than traditional forms of treatment? If it does, how much better? What are the costs & benefits of VR over traditional treatments? Also, we don't know much about long-term effects of using VR.
- Whatever endpoint is selected for the primary analysis, it should be clinically meaningful and reflect what is important to the patient.

Do you have recommendations for VR1 trial design considerations?

- Design considerations: Standard "needs analyses" – usually interviews. Then quick pilots, redesign and nailing down a protocol as much as it is possible, given that the tech changes every 6 months or so. Mixed methods approaches (interview pre- and post-VR plus user study) seem to always be crucial.
- Collaboration exercises: participatory design w/brainstorming & "cultural probes" where possible, focus groups where it's not possible. Also, it's important to involve expert "content providers" because the "look & feel" and ways of interacting in a virtual

environment can range pretty dramatically. Polling patient websites for specific conditions seems like an interesting and potentially important idea.

- My concern is the level of involvement of the patient population in the design. There should be clearly some input from end users in production of the interface. If the interface is unwieldy or not comfortable, it will not be used … The original proposal should be very clear on what is being tested for and expected end points of testing should be clearly defined. I think that different experimenters and experiments will need to consider the level of patient involvement in the original design of the test. My concern is that this is an important area where powerful biases can be introduced that can be continued through all levels of testing.
- I propose that the VR1 statement be modified to say that "consideration be given to allow potential patients or end user input in the design, but the experimenters should show discretion in the potential patients or end users amount of involvement in the development of the testing parameters".
- Patients are the ultimate customer of healthcare, and therapeutic VR is no different. The difference between a VR treatment and, say, a pharmacotherapy, is that patients are indeed knowledeable what they are looking for in a visualization and can provide immediate feedback in the design phase. In contrast, patients may not be experts in pharmacology, and may not be useful in the initial chemical compounding of pill (although they may well have comments later about the size, shape, color of the pill). The point is that VR treatments really need patient involvement early and often. The expert designing should take their feedback and work their magic from there, so it's not just patients who decide, but they must at least guide development.
- Although I do not oppose a patient centered design, I would broaden the language to include those who are working for/with patients. It may be difficult for true end users to have time/ability/access to the developers.
- I strongly advocate for patient and public involvement in design

Do you have recommendations for VR2 trial design considerations?

- Small pilot studies to verify whether or not the VR "works" -- in technical terms, in appropriateness (of content & interactions) and in terms of the protocol or treatment.
- The idea behind a VR2 design is to serve as a stepping stone between initial design, which happens in a lab-based setting, and the full-blown RCT, which is expensive and definitive. The VR2 is a "safe space" in the middle where the VR treatment resulting from VR1 is now inserted directly within a clinical environment, and handed right to the target patients for whom the treatment is intended. Then, the patients check out the equipment, try the experience, and provide feedback about whether it works, or doesn't work. Whether it's

comfortable, feasible, reasonable, etc. Also important to measure for early signs of tolerability / side effects (dizziness, fear, etc).

- VR2 is a very important stage. The technology is being tested and there is some measurement of the results. I suspect that because of the push to get products to market, most of the testing will be done here and there will not as much done at the VR3.
- VR2 should be focused on the "-ilities," as in "usability", "acceptability," "feasibility", and "tolerability." It's the first road test of the VR treatment in the context of the actual intended environment – a sort of ecological assessment in the "free range" clinical setting.
- I am not sure where retrospective reviews fall, but in addition to adding the intervention to a single arm, small population with active collection of adverse events, retrospective review of patients who have utilized the device should also be undertaken in addition to the prospective single arm intervention.
- Not only the hardware or the technology in general, the content design and strategies are very important to avoid these issues. This leads to what we discussed on standards for content development. This can be an opportunity to have that investigation on the side. I am not a medical doctor, but I would think different patients might react to VR in different ways, especially compared to healthy participants.
- I wonder if there will be different parameters for this given the platform? I remember someone telling me that IRB struggled with the idea that motion sickness was a possible adverse event. Do we have guidelines around adverse events in VR?

Do you have recommendations for VR3 trial design considerations?

- VR3 has some challenges. Again, it will be very challenging to perform a blinded study. I guess you could have a group use an interface that does nothing or gives erroneous results. There could be some ethical issues with that, especially if you performing this with a group of mentally ill patients. I suspect that this will be done mostly in aftermarket unless the investigator has the access to large scale support to get this done."
- Blinding should, at the very least, be achieved by blinding the analysts who are evaluating the final dataset. They do not need to know who is in which group, only that there are two (or more) groups that are labeled in the dataset. This is easy to achieve if carefully and meticulously done without contaminating the analysts.
- Concealment of allocation is very important but is rarely described. The issue is that patients randomized to VR may have a novelty effect and like the VR just because it's "cool," particularly when compared to something less "cool," like watching a 2D video, or TV, or whatever "less cool" control is employed. One approach is to NOT describe the two competing interventions at the time of content. Just say "we are comparing two different audiovisual experiences", or something like that.

- Properly powered is vital. There are so few RCTs at this level - most are underpowered. Cost is important.
- It's key to pre-define a clinically meaningful response. For PROs, this is considered the meaningful clinically important difference, or MCID. The MCID is sometimes known for a PRO, and sometimes not known. In latter case, many target a half standard deviation difference on the scale as evidence for an MCID, using the rules of Norman. Bottom line is this should be pre-specified, and the proportion benefiting, or P(B), between arms, should be compared and an NNT calculated.
- The primary outcome should compare the difference in difference (DID) in PROs before vs. after the received intervention, compared between groups. 95% CIs are standard and expected here as with any other RCT.
- How about starting to use other currently investigated technologies as the control? So instead of having regular patient vs VR-Patients, have for example tablet vs VR, or something like this.
- Although I fully agree, we found this to be an issue in our trials. Since we were the first to investigate the benefits of 360VR videos on patient information, anxiety reduction etc, calculating the power was a challenge
- Recommendations for patient monitoring: depends on the "hypothesized target of engagement" and hoped-for results of treatment or outcomes. For example, VR we develop for pain distraction for acute pain are VERY different for VR we develop for chronic pain management. And those are very different from VR we develop to help motivate rheumatoid arthritis patients to move or for people who are recovering from substance addictions to learn coping techniques when they encounter "triggers" that may lead to relapse. But in all cases, it's important to compare the data with traditional approaches.
- Similarly, assessment scores should have some relation to traditional assessment scores. In pain research, for example, there aren't many instruments that have been widely used for decades except the McGill Pain Questionnaire and arguably, one of several Visual Analog Scales & DNIC. Personally, for studies that are eventually intended for chronic pain patients, I don't think using methods of inflicting pain on "healthy subjects" is relevant.
- Just as with the VR2 studies, the VR3 should include clinically relevant PROs.
- Otherwise, we monitor patients via bio-sensors when that makes sense. Informally, we also observe how frequently they move & interact. We always ask in questionnaires if patients have experienced nausea (simsickness), but we also informally check that again observations. For example, in very early VR studies, the degree of simsickness was consistent with the relative change in stance. For instance, if a patient before the study maintained a 12" distance between their feet, and after the study maintain twice or three times that distance, you can be fairly certain they have some degree of simsickness.

- For pain distraction, we're conducting experiments with how, when & why attention should be directed. When VR pain distraction systems that have been successful for acute pain, for example, are used among chronic pain patients (CPP), patients often find them to be too stimulating or overwhelming. *It's possible to "dial down" those VEs*, however, by temporarily removing animations and certain sounds. (Dose de-escalation) In addition, the idea of "natural user interfaces" (NUIs) is that an application like VR can adapt to some of the specific needs of each user. So, for example, our low-level AI "reads" the streaming sensor data and "dials down" some of the stimuli in the pain distraction VR system. But it's very early days for this sort of thing.
- I'm only using this example as a concrete way to suggest that *some* aspects of VR studies can and should be standardized, but so much is possible that I'm wary about what, when & how we standardize. Seems like a moving target, at least re: technology. However, I do think that referring to traditional treatment assessments for specific health issues is necessary. Maybe that's the most important aspect to standardize. And I'd be wary of any NUI customization of any VR system, perhaps, until we get solid, replicable data about the outcomes of specific VR systems. (VR systems here refers to the technology, the content, the intended and possible interactions, and so on.)
- Stratifying patients is often accomplished by exclusions, at least in my domain. Since I'm interested in neuropathic pain that has CNS involvement, and because pain can be referred and complex, stratifying patients according to lower back pain and so on doesn't make sense -- unless someone is targeting that specifically. In other domains, however, I can imagine that stratifying patients would be productive.
